# Putative Circulating MicroRNAs Are Able to Identify Patients with Mitral Valve Prolapse and Severe Regurgitation

**DOI:** 10.3390/ijms22042102

**Published:** 2021-02-20

**Authors:** Paola Songia, Mattia Chiesa, Valentina Alfieri, Ilaria Massaiu, Donato Moschetta, Veronika Myasoedova, Vincenza Valerio, Laura Fusini, Paola Gripari, Marco Zanobini, Paolo Poggio

**Affiliations:** 1 Unit for the Study of Aortic, Valvular and Coronary Pathologies, Centro Cardiologico Monzino IRCCS, 20138 Milan, Italy; paola.songia@cardiologicomonzino.it (P.S.); valentina.alfieri@cardiologicomonzino.it (V.A.); ilaria.massaiu@cardiologicomonzino.it (I.M.); donato.moschetta@cardiologicomonzino.it (D.M.); veronika.myasoedova@cardiologicomonzino.it (V.M.); vincenza.valerio@cardiologicomonzino.it (V.V.); 2 Bioinformatics and Artificial Intelligence Facility (BioAI), Centro Cardiologico Monzino IRCCS, 20138 Milan, Italy; mattia.chiesa@cardiologicomonzino.it; 3 Dipartimento di Scienze Farmacologiche e Biomolecolari, Università degli Studi di Milano, 20133 Milan, Italy; 4 Dipartimento di Medicina Clinica e Chirurgia, Università degli Studi di Napoli Federico II, 80138 Naples, Italy; 5 Cardiovascular Imaging Department, Centro Cardiologico Monzino IRCCS, 20138 Milan, Italy; laura.fusini@cardiologicomonzino.it (L.F.); paola.gripari@cardiologicomonzino.it (P.G.); 6 Department of Cardiac Surgery, Centro Cardiologico Monzino IRCCS, 20138 Milan, Italy; marco.zanobini@cardiologicomonzino.it

**Keywords:** mitral valve disease, plasma, human, circulating signature, machine learning

## Abstract

Mitral valve prolapse (MVP) associated with severe mitral regurgitation is a debilitating disease with no pharmacological therapies available. MicroRNAs (miRNA) represent an emerging class of circulating biomarkers that have never been evaluated in MVP human plasma. Our aim was to identify a possible miRNA signature that is able to discriminate MVP patients from healthy subjects (CTRL) and to shed light on the putative altered molecular pathways in MVP. We evaluated a plasma miRNA profile using Human MicroRNA Card A followed by real-time PCR validations. In addition, to assess the discriminative power of selected miRNAs, we implemented a machine learning analysis. MiRNA profiling and validations revealed that miR-140-3p, 150-5p, 210-3p, 451a, and 487a-3p were significantly upregulated in MVP, while miR-223-3p, 323a-3p, 340-5p, and 361-5p were significantly downregulated in MVP compared to CTRL (*p* ≤ 0.01). Functional analysis identified several biological processes possible linked to MVP. In addition, machine learning analysis correctly classified MVP patients from CTRL with high accuracy (0.93) and an area under the receiving operator characteristic curve (AUC) of 0.97. To the best of our knowledge, this is the first study performed on human plasma, showing a strong association between miRNAs and MVP. Thus, a circulating molecular signature could be used as a first-line, fast, and cheap screening tool for MVP identification.

## 1. Introduction

Mitral valve prolapse (MVP) is a debilitating disease that, to date, has affected more than 176 million people worldwide with a prevalence of 2–3% in the general population [1,2]. The main cause of MVP is an alteration of the well-organized leaflet structure due to a myxomatous degeneration, which is characterized by distinctive histological changes with elongated and redundant chordal apparatus [3]. Based on clinical patterns, echocardiographic findings, and gross surgical appearances, the degenerative mitral valve disease has been divided into Barlow’s disease (BW) and fibro-elastic deficiency (FED), which were originally described by Carpentier [4,5,6]. An excessive and diffuse accumulation of glycosaminoglycans is the main feature of BW disease, whereas FED is characterized by extremely thin leaflets and chordae [7]. Up to now, two-dimensional (2D) echocardiography represents the benchmark for MVP diagnosis and the assessment of the disease severity [8,9]. In addition, the combination of 2D with 3D echocardiography provides detailed morphological and functional assessment [10,11,12,13]. Unfortunately, there are no medical therapies able to prevent or treat patients affected by this frequent pathology; therefore, the surgical or, more recently, percutaneous interventions are the only available options when the prolapse causes severe regurgitation and symptoms occur [8]. 

In clinical practice, biomarkers represent an important tool for a better diagnosis and prognosis of a specific pathological condition [14]. To date, there are no specific circulating biomarkers for MVP identification. In this context, researchers have found a possible association between osteoprotegerin [15,16], haptoglobin, platelet basic protein, complement component C4b levels [17], and MVP. However, none of these biomarkers is specific for the pathology.

Recently, a new class of circulating biomarkers, called microRNAs (miRNAs), has emerged. MiRNAs are short noncoding RNAs, which negatively regulate gene expression at the post-transcriptional level by inhibiting the protein translation or promoting the mature RNA (mRNA) degradation [18]. In the last few years, the role of miRNAs has been assessed in different pathological conditions including cardiovascular diseases (e.g., coronary artery diseases, cardiomyopathy, myocardial infarction, and aortic valve stenosis) [19,20,21,22,23]. Concerning MVP, two studies evaluated circulating miRNAs in animal models [24,25], and recently, Bulent Vatan et al. [26] analyzed plasma miRNAs in patients with mitral chordae tendineae rupture, which is a condition closely linked to MVP but not necessarily to myxomatous degeneration. Thus, our aim was to evaluate the circulating miRNA profile in human MVP, identifying a possible miRNA signature that is able to discriminate MVP patients from healthy subjects with high accuracy and shed light on putative altered molecular pathways in MVP.

## 2. Results

### 2.1. Circulating miRNA Linked to Mitral Valve Prolapse

**Figure 1:**
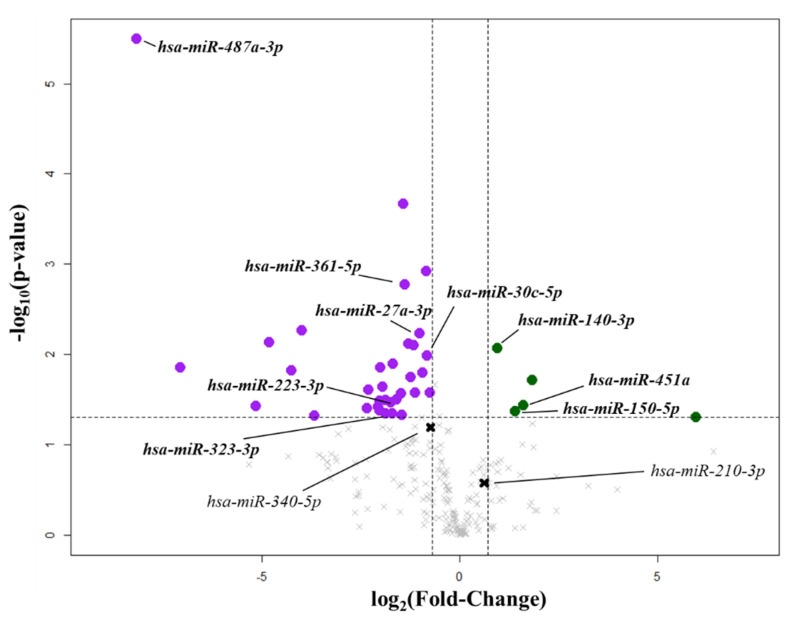
Circulating microRNAs (miRNA) linked to mitral valve prolapse. Volcano plot represents the differential expression of circulating miRNAs between healthy subjects (CTRL; *n* = 4) and mitral valve prolapsed patients (MVP; *n* = 4). The vertical lines correspond to fold changes of −0.7 (downregulation) and +0.7 (upregulation). The horizontal lines indicate a *p*-value of 0.05. The purple points denote downregulated miRNAs, while the green points denote the upregulated miRNAs with statistical significance in comparison to healthy subjects. The bold black crosses depict not differentially expressed miRNAs investigated in the validation phase.

To investigate possible differences between MVP patients (*n* = 4) and matched healthy subjects (CTRL; *n* = 4), we conducted an initial screening of 384 miRNAs. Out of the 384 miRNAs screened, 201 were detectable in both groups. We identified forty miRNAs differentially expressed between the two cohorts (*p* < 0.05; Table S1). In particular, five miRNAs were upregulated, while thirty-five were downregulated in MVP patients compared to CTRL (Figure 1).

### 2.2. Validation Phase

Following the screening phase, we performed the real-time PCR (qPCR) in a larger cohort of MVP patients (*n* = 43) and CTRL (*n* = 34). We analyzed nine interesting miRNAs differentially expressed between the two groups, as well as two not significantly different miRNAs, such as miR-340-5p and miR-210-3p, which are known to be involved in other cardiovascular diseases. In particular, platelet-derived miR-340-5p is upregulated in patients with coronary artery disease as compared to healthy controls [27]. Meanwhile, in other pathological conditions, such as ischemic stroke, miR-340-5p appears to be downregulated [28]. Regarding miR-210-3p, researchers have reported its involvement in atherosclerosis, acute coronary syndrome, valvular heart diseases, and pulmonary arterial hypertension [29].

**Figure 2:**
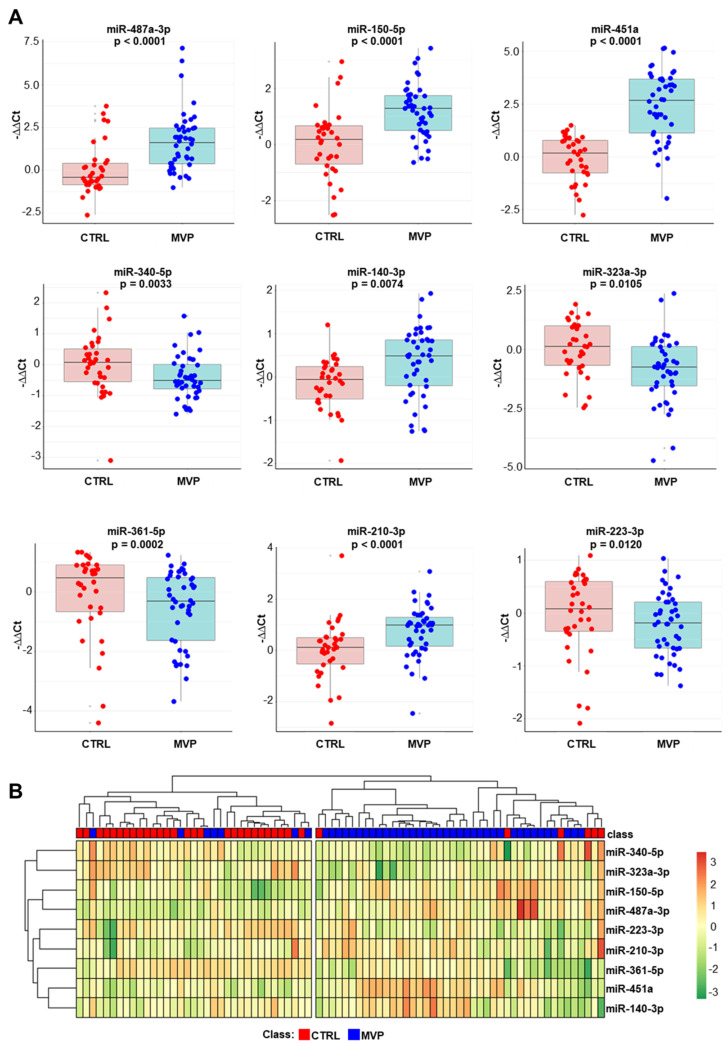
Circulating miRNA validation. (**A**) Validation analysis confirmed that miR-487a-3p, miR-150-5p, miR-140-3p, miR-451a, and miR-210-3p were significantly upregulated in the mitral valve prolapsed group (MVP, *n* = 43) compared with healthy subjects (CTRL, *n* = 34). Instead, miR-340-5p, miR-323a-3p, miR-361-5p, and miR-223-3p were significantly downregulated in the MVP group compared with the CTRL group. Data are depicted as box-and-whisker plots of –delta–delta cycle threshold (−ΔΔ*C*t). (**B**) The heatmap shows that the nine miRNAs differentially expressed in both the screening and the validation cohorts discriminated healthy subjects (CTRL; red squares) from patients with mitral valve prolapse (MVP; blue squares). MiRNAs expression levels were expressed as standardized values and displayed as a color gradient from dark red (i.e., highest expression level) to dark green (i.e., lowest expression level).

Our results confirmed that nine miRNAs were statistically different in the MVP group in comparison to CTRL subjects (Figure 2A), while two miRNAs were similar in the two cohorts (Figure S1). However, we found that miR-487a-3p was downregulated in the screening phase, while it was upregulated in the validation phase. This discrepancy could be explained by the small sample size of the screening phase. This is corroborated by the log2 fold change (log_2_FCs) of the validated miRNAs calculated in the screening and the validation sets (Figure S2). When miR-487a-3p was included in this analysis, there was no correlation between the two techniques (R = 0.01, *p* = 0.975; Figure S2A). However, a strong correlation between the two techniques was obtained when miR-487a-3p was excluded (R = 0.92, *p* = 0.0005; Figure S2B). In addition, we performed an unsupervised hierarchical clustering analysis, generating a specific heatmap (Figure 2B). The results underlined that all validated miRNAs allow us to discriminate MVP and CTRL.

### 2.3. Functional Analysis

To gain further insights into the potential biological functions of these nine miRNAs, we applied a guilt-by-association in silico approach [30] to identify which genes, cell types, and tissues were potentially involved in MVP pathophysiology.

**Figure 3:**
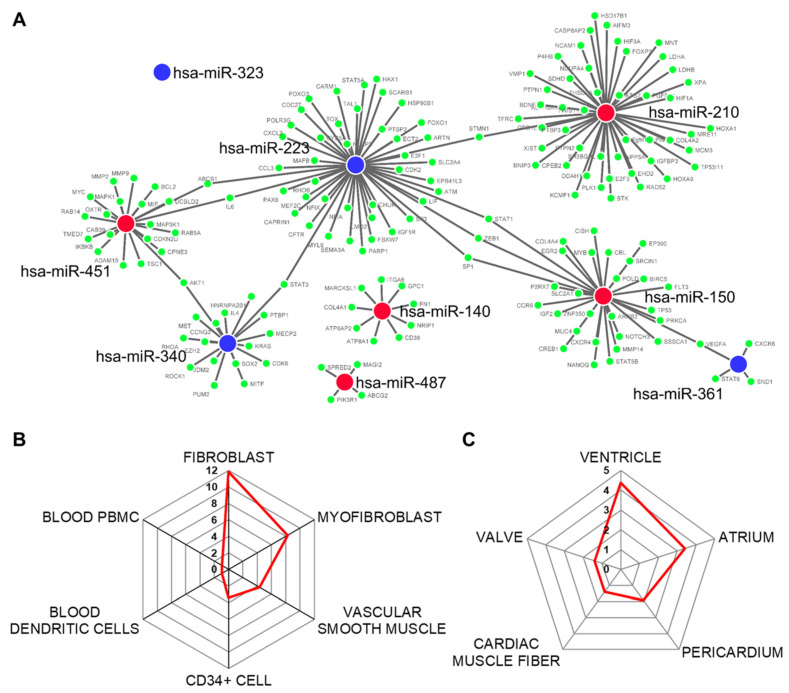
miRNA–mRNA target prediction. (**A**) mRNA transcripts potentially modulated by the nine differentially expressed miRNAs between healthy subjects (CTRL) and mitral valve prolapsed patients (MVP). Enrichment analysis showing which cell types (**B**) and tissues (**C**) express the modulated mRNA transcripts. The axis values represent the combined score computed by taking the log of the *p*-value from the Fisher exact test and multiplying that by the z-score of the deviation from the expected rank.

MiRNA–mRNA target prediction revealed that 171 genes are potentially modulated by the nine differentially expressed miRNAs (Figure 3A). However, since we took into account only experimental validated interactions and miR-323 did not have any, it was excluded in this analysis. The modulated mRNAs are mainly represented in fibroblast and myofibroblast and in vascular smooth muscle and CD34+ cells to a lesser extent (Figure 3B). Regarding the location of these cells, the main cardiovascular tissues involved were the heart ventricles and atriums, the pericardium, and the cardiac muscle fibers as well as the heart valves (Figure 3C).

**Figure 4:**
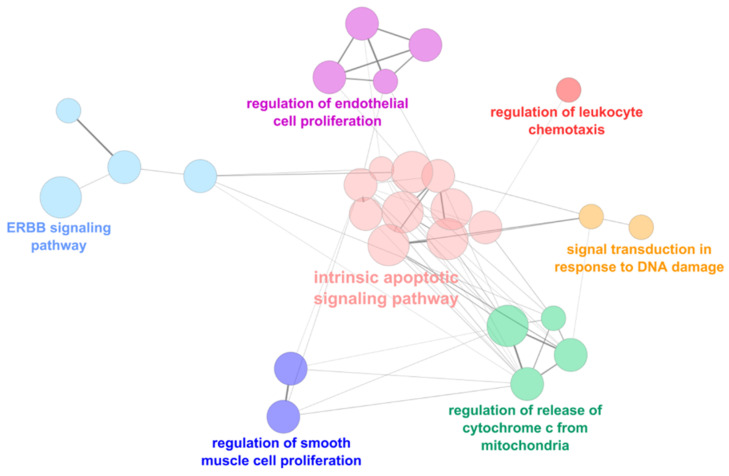
Functional analysis of the upregulated miRNAs. Signaling pathways directly modulated by upregulated miRNAs in mitral valve prolapsed patients (MVP) in comparison to healthy subjects (CTRL). The node color indicates different pathways while node size is proportional to the gene-set size. Edge thickness is proportional to the similarity between two gene-sets. ERBB: erythroblastic oncogene B.

The functional analyses showed that genes regulated by these miRNAs are implicated in several cellular processes. In particular, the main downregulated processes, identified by the upregulated miRNAs, were the intrinsic apoptotic signaling pathways, the regulation of endothelial and smooth muscle cell proliferation, the signal transduction in response to DNA damage, and the erythroblastic oncogene B (ERBB) signaling pathway (Figure 4).

**Figure 5:**
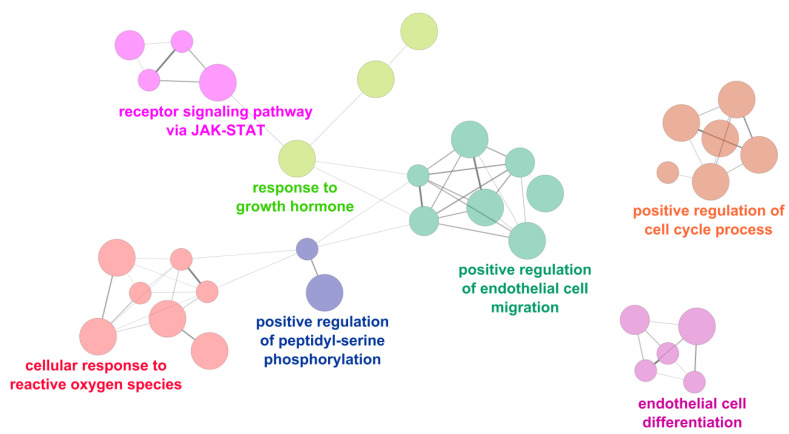
Functional analysis of the downregulated miRNAs. Signaling pathways directly modulated by downregulated miRNAs in mitral valve prolapsed patients (MVP) in comparison to healthy subjects (CTRL). The node color indicates different pathways, while the node size is proportional to the gene-set size. Edge thickness is proportional to the similarity between two gene sets. JAK-STAT: janus kinase-signal transducer and activator of transcription protein.

Conversely, the main upregulated processes were the cellular response to reactive oxygen species, the receptor signaling pathway via janus kinase-signal transducer and activator of transcription protein (JAK-STAT), and the regulation of endothelial cell migration and differentiation (Figure 5).

### 2.4. Mitral Valve Prolapse Circulating miRNA Signature Strength

**Figure 6:**
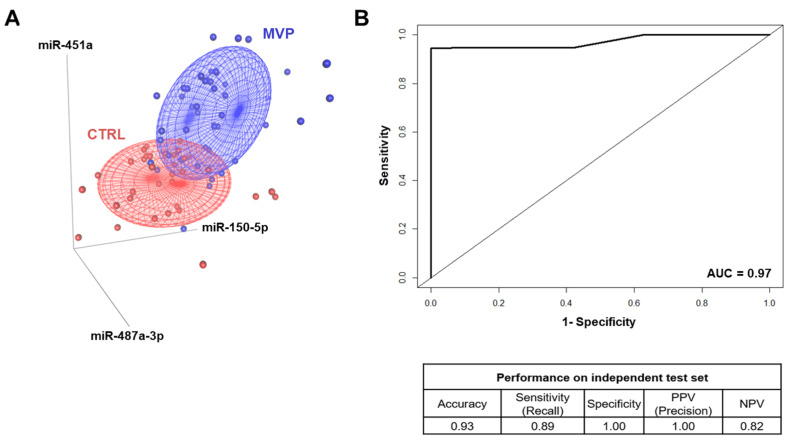
Mitral valve prolapse circulating miRNA signature robustness. (**A**) Scatter plot based on the most statistically significant (*p* < 0.001) miRNAs: miR-150-5p, -451a, and -487a-3p. The red dots represent healthy subjects (CTRL), while the blue dots represent the mitral valve prolapsed patients (MVP). (**B**) Performances of the classification model on an independent test set in terms of accuracy, specificity, sensitivity (i.e., recall), positive predictive value (PPV), negative predictive value (NPV; i.e., precision), and the area under the receiving operator characteristic (AUC) curve.

Among the validated miRNAs, we further selected the most statistically significant miRNAs (*p*-value < 0.001), namely miR-150-5p, miR-451a, and miR-487a-3p, and we assessed their capability to identify MVP patients and CTRL as two distinct groups. The 3D scatter plot in Figure 6A underlined that these miRNAs allowed us to differentiate MVP patients from CTRL. Furthermore, we performed a machine learning analysis, and taking into account the prediction model, we were able to correctly classify 93% of samples belonging to the independent test set with an area under the receiving operator characteristic (AUC) curve of 0.97, a sensitivity of 0.89, and a specificity equal to 1 (Figure 6B). Together, these data highlighted that the model based on miR-150-5p, -451a, and -487a-3p correctly identified MVP patients from CTRL with high accuracy. To ensure that the miRNA signature was specific to MVP and not secondary to other differences between MVP patients and CTRL, we performed a logistic regression associating the model comprising the three miRNAs to the class of interest (MVP vs. CTRL) and to the variables significantly related with the disease (see patient population section); namely, sex, hypertension, angiotensin-converting enzyme (ACE) inhibitors, and beta-blocker therapy. This analysis suggests a strong association of the three miRNAs with MVP (*p* = 0.0011), while there was no significant association considering sex (*p* = 0.16), hypertension (*p* = 0.28), ACE inhibitors (*p* = 0.47), nor beta-blocker therapy (*p* = 0.15).

Furthermore, considering the two MVP subgroups (fibro-elastic deficiency, FED and Barlow’s disease, BW; for patients characteristics, see Table S2), differential analysis underlined that miR-150-5p represented the only miRNA statistically different between FED and BW patients (Table 1).

## 3. Discussion

To the best of our knowledge, this is the first study performed on human plasma from MVP patients, showing a strong association between several circulating miRNAs and MVP pathology. The myxomatous degeneration of the mitral valve is the most common cause of mitral valve prolapse, which required surgical intervention when the prolapse causes severe regurgitation and symptoms occur [8]. Echocardiography is the only clinical reliable tool for MVP diagnosis, and circulating biomarkers could provide valuable insights into MVP etiology as well as patient stratification. In this context, Deroyer et al. [31] showed that apolipoprotein-A1 was an independent predictor of mitral regurgitation (MR) severity. In addition, in a comparative proteomic study [17], the authors underlined reduced plasma levels of haptoglobin, platelet basic protein, and complement component C4b in the MVP patients with MR compared to matched control cases. However, the clinical relevance is unclear, in part because most of the identified biomarkers had moderate AUC. Recently, our group showed an altered systemic oxidative stress homeostasis as well as increased osteoprotegerin (OPG) plasma levels in MVP patients [15]. In addition, in a multivariable regression model combining OPG with oxidative stress markers, we were able to discriminate MVP patients from healthy subjects with high accuracy and precision [16]. However, none of these biomarkers has a high specificity for the MVP pathology.

In the last years, microRNAs represent an emerging class of circulating biomarkers widely studied in different pathological conditions including cardiovascular diseases [19,20,21,22,23]. Concerning MVP, a limited number of studies investigated circulating microRNAs but only in animal models. In particular, Hulanicka et al. [24] analyzed the miRNAs expression in the plasma of Dachshunds with myxomatous mitral valve disease (MMVD). They identified downregulation of two miRNAs (cfa-miR-30b and cfa-miR-133b) that regulate connective tissue growth factor, which is a key molecule in fibrotic processes linked to canine mitral valve diseases development and progression. A second study reported that eleven miRNAs were differentially expressed in the serum of dogs at a different stage of MMVD and congestive heart failure (CHF) compared to normal dogs [25]. Interestingly, the miRNA expression changes were greater as disease severity progressed. An additional study, through next-generation sequencing, highlighted eight circulating miRNAs differentially expressed between normal dogs and dogs with CHF secondary to MMVD [32].

Concerning human studies, Bulent Vatan et al. [26] evaluated plasma miRNA expression in patients with mitral chordae tendineae rupture (MCTR) without MVP. Researchers have reported downregulation of twenty-two miRNAs in MCTR patients in comparison to control subjects. In addition, the putative targets of these microRNAs were related to the MCTR pathophysiology. Our study revealed a different miRNA plasma profile with nine validated miRNAs differentially expressed between MVP patients and healthy subjects. In both studies, miR-150-5p was significantly upregulated in patients compared to the respective controls. However, miR-223-3p was statistically different between patients and controls but in the opposite direction. The differences are probably due to the patient population enrolled in each study. Indeed, we took into consideration posterior MVP patients who underwent surgical procedure due to severe regurgitation, regardless of chordae rupture. Nonetheless, considering our results, we were able to develop a putative circulating microRNA signature, taking into account three miRNAs (miR-150-5p, -451a, and -487a-3p). 

Another important issue that should be taken into account is the role of biomechanics in determining chordae rupture and anterior, posterior, or both leaflets prolapse. Several studies have performed finite element analysis evaluating mechanical forces interplay [33,34]. However, the correlation between these forces and circulating miRNAs has not been investigated yet. Thus, future studies focusing on these particular aspects could unveil biological insights directly linked to normal and pathological forces on the mitral valve structure. 

In our study, cell type enrichment analysis, based on validated miRNAs, recognized specific cell populations belonging to different cardiovascular tissues, including the mitral valve. In addition, the functional analysis underlined distinct pathways associated with MVP. In particular, we identified well-characterized signaling pathways such as endothelial cell migration and proliferation [15,35,36], cell response to oxygen reactive species [15], and deregulation of the extracellular matrix homeostasis [37,38,39]. Instead, the newly identified erythroblastic oncogene B (ERBB) and janus kinase-signal transducer and activator of transcription protein (JAK-STAT) signaling pathways could unveil new mechanisms involved in MVP progression.

Based on echocardiographic findings, degenerative mitral valve disease can be classified into myxomatous MVP, alternatively known as Barlow’s disease (BW), and fibro-elastic deficiency (FED). Chen et al. [40] identified a cluster of tissue miRNAs that differentially express between BW and FED with putative target genes crucial for valvular extracellular matrix homeostasis. Our results can indicate that distinct molecular mechanisms are implicated in BW and FED pathophysiology and it could be identified in the systemic circulation. Indeed, we found that FED and BW patients showed a different expression of miR-150-5p, which is known to be involved in several processes, including proliferation [41,42,43].

This study has different limitations. First, in the validation phase, we have not evaluated possible differences in terms of miRNA expression between FED and BW patients, since the subject number in the two MVP subgroups is limited. Second, for the same reason, we could not investigate if the identified classification model is able to distinguish BW patients from FED. Lastly, our cohort is based on patients eligible for mitral valve surgery with MVP and severe regurgitation. Thus, we did not assess if our miRNA signature is able to identify patients with MVP and mild or moderate mitral regurgitation.

## 4. Materials and Methods

### 4.1. Patient Population

The study was approved by the Institutional Review Board and by the Ethical Committee of Centro Cardiologico Monzino (IRCCS) in accordance with the principles outlined in the Declaration of Helsinki (1964). Written informed consent to participate in this study was obtained from all the participants. 

Preoperative inclusion criteria were the need for an elective, isolated surgical procedure, over 18 years of age, an ejection fraction of >30%, normal sinus rhythm, and no history of atrial fibrillation. Exclusion criteria were the presence of a bicuspid aortic valve, premature menopause and/or osteoporosis, prior aortic or mitral valve surgery, rheumatic heart disease, endocarditis, active malignancy, chronic liver failure, calcium regulation disorders (hyperparathyroidism, hyperthyroidism, and hypothyroidism), and chronic or acute inflammatory states (sepsis, autoimmune disease, and inflammatory bowel disease). Forty-three patients, requiring mitral valve replacement due to posterior MVP with severe regurgitation, were enrolled in the study. In all patients, blood collection was performed before coronary angiography. Age-matched CTRL (*n* = 34) with normal sinus rhythm, no electrocardiographic alterations, and no history of atrial fibrillation were screened from those referred to Centro Cardiologico Monzino, IRCCS for cardiovascular health-screening evaluation. Blood samples were collected at a scheduled visit. The demographic and clinical features of the two study groups are listed in Table 2. 

### 4.2. Blood Sampling

Peripheral blood samples were drawn from patients and healthy subjects while fasting into tubes containing ethylenediaminetetraacetic acid (EDTA). Anti-coagulated blood was centrifuged at 2000× *g* for 10 min at 4 °C within 15 min after being drawn. Plasma was separated, and aliquots were stored at −80 °C until analysis.

### 4.3. TaqMan Human miRNA Card A Arrays

RNA extraction was performed from plasma using the Total RNA Purification Plus Kit (Norgen Biotek Corp., Thorold, ON, Canada) according to the manufacturer’s instructions. The TaqMan Human microRNA Card A Arrays version 3.0 (Thermo Fisher Scientific, Waltham, MA, USA) was used for evaluating the expression of a total of 384 miRNAs. The megaplex pool primers were used for reverse transcription (RT), pre-, and amplification steps and performed according to the manufacturer’s protocol on a 7900HT Real-Time PCR System (Thermo Fisher Scientific, Waltham, MA, USA). Then, 350 ng of RNA were retro-transcribed with 40 cycles at 16 °C for 2 min, 42 °C for 1 min, and 50 °C for 1 s, followed by incubation at 85 °C for 5 min. The pre-amplification was performed by a serial incubation at 95 °C for 10 min, 55 °C for 2 min, 72 °C for 2 min, 12 cycles at 95 °C for 15 s and 60 °C for 4 min, followed by incubation at 99.9 °C for 10 min. Finally, the samples were loaded into the run plate and incubated at 50 °C for 2 min, 94.5 °C for 10 min, 40 cycles at 97 °C for 30 s, and 59.7 °C for 1 min.

We filtered out miRNA that did not pass the quality controls. The excluded miRNAs had one or more of the following features: low signal in linear phase; bad passive reference signal (ROX); low quantification cycle (Cq) confidence; threshold cycle (Ct) algorithm failed; exponential algorithm failed; and thresholding algorithm failed. In addition, we considered only miRNAs that were expressed and passed the quality tests in all the samples. miR-186-5p was used as a housekeeping gene. The expression value of each miRNA is reported as log2 fold change (logFC) considering relevant the log2 fold change greater or lower than 0.7 compared to healthy subjects and a *p*-value < 0.05.

### 4.4. Reverse Transcription and Real-Time PCR

Total RNA was converted into cDNA using a TaqMan Advanced miRNA cDNA Synthesis Kit (Thermo Fisher Scientific, Waltham, MA, USA) followed by an amplification step according to the manufacturer’s protocol. This synthesis kit uses a universal reverse transcription (RT) chemistry to prepare the cDNA template for use with TaqMan™ Advanced miRNA Assays (Thermo Fisher Scientific, Waltham, MA, USA) for the detection and quantification of mature miRNAs in biological samples. The specific assays’ identification (ID) are reported in Table S3. qPCR was carried out on an ABI Prism 7900 HT (Thermo Fisher Scientific, Waltham, MA, USA), according to the manufacturer’s instructions, and analysis was performed using software SDS2.4 (Thermo Fisher Scientific, Waltham, MA, USA). miR-186-5p was used as a housekeeping gene and the data were reported as logFC.

### 4.5. MiRNA–mRNA Target Prediction

To evaluate miRNA–mRNA interactions, we used CyTargetLinker [44], which is a Cytoscape (v3.7.1) [45] plug-in that builds complex miRNA–mRNA association networks, exploiting the ‘miRTarBase’ database [46]. To be as reliable and conservative as possible, we discarded mRNA targets without “strong experimental evidence”.

### 4.6. Functional and Cell Type Enrichment Analyses

The predicted mRNA targets were fed into ClueGO [47], which is a Cytoscape app that performs the function analysis on a pre-selected set of genes, exploiting the hypergeometric test. The Gene Ontology Biological Processes (GO-BP) database has been selected as the reference [48]. Finally, in order to assess which tissues and cell types are potentially involved in the pathophysiology, the web application ‘EnrichR’ was used to perform enrichment analysis, exploiting the ARCHS4 repository [49]. Pathways with an associated *p*-value < 0.05 were deemed as significant.

### 4.7. Machine Learning Analysis

To assess the discriminative power of selected miRNAs, we implemented a supervised machine learning analysis. First, we randomly split the dataset (43 MVP and 34 CTRL) into two subsets: a balanced set (24 MVP and 25 CTRL), called the “learning dataset”, and an “independent test set” (19 MVP and 9 CTRL). Then, we applied a 5-fold cross-validation strategy to the learning dataset that was iteratively divided into training sets (19 MVP and 20 CTRL), which were used to build a random forest classifier, and into validation sets (5 MVP and 5 CTRL), which were used to test the classifier performance. For each fold, the accuracy, specificity, sensitivity (i.e., recall), positive predictive value (PPV), negative predictive value (NPV; i.e., precision), and the area under the receiving operator characteristic (ROC) curve (AUC) were calculated for each validation set [50]. Finally, based on the highest AUC, we chose the best model (i.e., the best combination of hyperparameters) that was successively tested on the independent test set, measuring accuracy, specificity, sensitivity, PPV, NPV, and AUC [51]. The analysis was implemented exploiting the ‘ROCR’, ‘caret’, ‘randomForest’, ‘car’, and ‘cluster’ R packages.

### 4.8. Statistical Analysis

Statistical analysis was accomplished by implementing the Shapiro–Wilk test to assess the normality of the data distributions and the two-tailed Student’s t-test in R environment (http://www.R-project.org/) as well as in Prism (GraphPad 7). Differences were deemed significant if the *p*-value was < 0.05. We developed the heat map performing an unsupervised hierarchical clustering analysis based on validated miRNAs. The correlation distance matrix was calculated to determine the clustering distance, and the hierarchical clustering method was the ‘Average Linkage’.

## 5. Conclusions

To the best of our knowledge, this is the first study performed on human plasma obtained from posterior MVP patients, showing a strong association between miRNAs and MVP pathology. Further studies are required to understand if the identified signaling pathways directly modulate the MVP pathophysiology. In addition, other studies are needed to identify the possible role of circulating miR-150-5p in order to evaluate its causal relationship with different MVP pathophysiology (FED and BW). 

Taken together, these data (1) open new possibilities that could allow us to identify new possible pharmacological targets to slow down or even halt MVP progression and (2) indicate that circulating molecular signatures could be identified and possibly used in clinical practice as a first-line, fast, and cheap screening tool for MVP patients’ identification.

## Figures and Tables

**Table 1:** miRNA expression levels in FED and BW patients.

FED vs. BW		
miRNAs	logFC	*p*-value
miR-140-3p	0.12	0.74
miR-150-5p	−1.2	0.03
miR-210-3p	1	0.1
miR-223-3p	0.29	0.39
miR-27a-3p	0.11	0.87
miR-30c-5p	0.22	0.56
miR-323a-3p	−0.59	0.48
miR-340-5p	−0.16	0.71
miR-361-5p	−0.42	0.43
miR-451a	−1.07	0.1
miR-487a-3p	0.41	0.6

BW: Barlow’s disease patients; FED: fibro-elastic deficiency patients; logFC: log2 fold change.

**Table 2:** Demographic and clinical variables of MVP patients and CTRL subjects.

Variables	CTRL (*n* = 34)	MVP (*n* = 43)	*p*-Value
Age (years)	53.5 ± 9.4	54.3 ± 9.9	0.701
Male subjects, *n* (%)	16 (47)	34 (79)	0.004
BMI	25.2 ± 4.9	24.4 ± 3.3	0.390
Diabetes, *n* (%)	2 (6)	-	-
Hypertension, *n* (%)	5 (15)	16 (37)	0.039
Dysplidemia *n* (%)	15 (44)	19 (44)	1.000
Smokers	5 (15)	13 (30)	0.175
Total Cholesterol (mg/dL)	215.4 ± 45.9	202.5 ± 34.2	0.203
Triglycerides (mg/dL)	114.9 ± 50.6	102.4 ± 52.5	0.317
HDL (mg/dL)	65.3 ± 36.0	58.0 ± 13.5	0.315
LDL (mg/dL)	129.4 ± 38.5	124.3 ± 32.5	0.568
Drug Therapies			
Antiplatelets, *n* (%)	2 (6)	6 (14)	0.291
Angiotensin II receptor blockers, *n* (%)	1 (3)	5 (12)	0.220
Angiotensin-converting enzyme inhibitors, *n* (%)	1 (3)	13 (30)	0.002
Calcium channel blockers, *n* (%)	1 (3)	1 (2)	1.000
Beta-blockers, *n* (%)	3 (9)	13 (30)	0.026
Statins, *n* (%)	3 (9)	6 (14)	0.723
Echocardiographic data			
LVEF (%)	62.5 ± 8.3	63.8 ± 6.2	0.465
Left Ventricular Diastolic Volume (mL)	96.8 ± 28.5	146.0 ± 52.5	<0.001
Left Ventricular Systolic Volume (mL)	37.0 ± 17.3	41.9 ± 16.2	<0.001
Left Atrial Area (cm^2^)	18.2 ± 3.9	28.0 ± 7.0	<0.001
PAPs	26.4 ± 3.7	34.5 ± 7.9	<0.001
EROA (cm^2^)	-	0.5 ± 0.2	-

Values are mean ± SD or *n* (%). CTRL: healthy subjects; EROA: effective regurgitant orifice area; LVEF: left ventricular ejection fraction; MVP: mitral valve prolapse patients; PAPs: pulmonary artery systolic pressure.

## Data Availability

The datasets used and analyzed during the current study are available from the corresponding author on reasonable request.

## Supplementary Materials

The following are available online at https://www.mdpi.com/1422-0067/22/4/2102/s1, Supplementary Table S1. miRNAs differentially expressed in the screening phase between MVP patients and healthy subjects. Supplementary Table S2. Demographic and clinical variables of BW and FED patients. Supplementary Table S3. TaqMan probe assays’ ID. Supplementary Figure S1. Quantitative reverse transcription polymerase chain reaction validation. Supplementary Figure S2. Person’s correlation of the log2FCs for the validated miRNAs between the screening and validation phases.
