# Supplementary material for: Putative Circulating MicroRNAs Are Able to Identify Patients with Mitral Valve Prolapse and Severe Regurgitation
Source: Int J Mol Sci. 2021 Feb 20;22(4):2102. doi: 10.3390/ijms22042102 (PMC7924183; doi:10.3390/ijms22042102)
Supplement: Supplementary file 1 [file ijms-22-02102-s001.pdf]

Putative Circulating MicroRNAs Are Able to Identify Patients with Mitral Valve Prolapse and Severe Regurgitation

Paola Songia [1], Mattia Chiesa [2], Valentina Alfieri [1], Ilaria Massaiu [1], Donato Moschetta [1,3], Veronika Myasoedova [1], Vincenza Valerio [1,4], Laura Fusini [5], Paola Gripari [5], Marco Zanobini [6] and Paolo Poggio [1,*]

1 Unit for the Study of Aortic, Valvular and Coronary Pathologies, Centro Cardiologico Monzino IRCCS, 20138 Milan, Italy; paola.songia@cardiologicomonzino.it (P.S.); valentina.alfieri@cardiologicomonzino.it (V.A.); ilaria.massaiu@cardiologicomonzino.it (I.M.); donato.moschetta@cardiologicomonzino.it (D.M.); veronika.myasoedova@cardiologicomonzino.it (V.A.M.); vincenza.valerio@cardiologicomonzino.it (V.V.)
2 Bioinformatics and Artificial Intelligence facility (BioAI), Centro Cardiologico Monzino IRCCS, 20138 Milan, Italy; mattia.chiesa@cardiologicomonzino.it (M.C.)
3 Dipartimento di Scienze Farmacologiche e Biomolecolari, Università degli Studi di Milano, 20133 Milan, Italy
4 Dipartimento di Medicina Clinica e Chirurgia, Università degli Studi di Napoli Federico II, 80138 Naples, Italy
5 Cardiovascular Imaging Department, Centro Cardiologico Monzino IRCCS, 20138 Milan, Italy, laura.fusini@cardiologicomonzino.it (L.F.); paola.gripari@cardiologicomonzino.it (P.G.)
6 Department of Cardiac Surgery, Centro Cardiologico Monzino IRCCS, 20138 Milan, Italy, marco.zanobini@cardiologicomonzino.it (M.Z.)

* Correspondence: paolo.poggio@ccfm.it; Tel.: +390258002853

Supplementary Tables

Supplementary Table S1. miRNAs differentially expressed in the screening phase between MVP patients and healthy subjects.

miRNA	logFC	*p*-value	miRNA	logFC	*p*-value
hsa-miR-487	-8.16	0.000003	hsa-miR-376a	-2.30	0.0245
hsa-miR-331-3p	-1.43	0.0002	hsa-miR-223-3p	-0.75	0.0264
hsa-miR-374a	-0.85	0.0012	hsa-miR-339-5p	-1.13	0.0268
hsa-miR-361-5p	-1.39	0.0017	hsa-let-7a	-1.49	0.0274
hsa-miR-654-5p	-3.99	0.0054	hsa-miR-127-3p	-1.60	0.0318
hsa-miR-27a	-1.02	0.0058	hsa-miR-370	-1.88	0.0321
hsa-miR-362-3p	-4.81	0.0073	hsa-miR-379	-2.02	0.0326
hsa-miR-27b	-1.29	0.0077	hsa-miR-323-3p	-1.75	0.0340
hsa-miR-130a	-1.15	0.0079	hsa-miR-451	1.61	0.0365
hsa-miR-140-3p	0.96	0.0086	hsa-miR-576-5p	-5.15	0.0372
hsa-miR-30b	-0.82	0.0104	hsa-miR-433	-2.06	0.0383
hsa-miR-181a	-1.69	0.0128	hsa-miR-431	-2.34	0.0400
hsa-miR-891a	-7.06	0.0139	hsa-miR-410	-2.02	0.0418
hsa-miR-889	-2.00	0.0141	hsa-miR-150	1.39	0.0424
hsa-miR-485-5p	-4.25	0.0151	hsa-miR-494	-1.87	0.0449
hsa-miR-30c	-0.94	0.0161	hsa-miR-495	-1.71	0.0454
hsa-miR-324-5p	-1.23	0.0178	hsa-miR-9	-1.46	0.0469
hsa-miR-501-3p	1.83	0.0192	hsa-miR-374b	-0.52	0.0480
hsa-miR-15b	-0.64	0.0216	hsa-miR-199b-5p	-3.67	0.0481
hsa-miR-376c	-1.96	0.0228	hsa-miR-184	5.96	0.0500

Supplementary Table S2. Demographic and clinical variables of BW and FED patients.

Variables	BW (n = 27)	FED (n = 16)	p-value
Age (years)	50.3 ± 9.1	61.1 ± 7.2	< 0.001
Male subjects, n (%)	20 (74%)	14 (88%)	0.446
BMI	23.7 ± 3.1	25.6 ± 3.4	0.069
Diabetes, n (%)	-	-	
Hypertension, n (%)	8 (30%)	8 (50%)	0.209
Dysplidemia n (%)	10 (37%)	9 (56%)	0.341
Smokers	8 (30%)	5 (31%)	1.000
Total Cholesterol (mg/dL)	200.4 ± 35.2	206.1 ± 32.5	0.599
Triglycerides (mg/dL)	95.1 ± 37.7	114.8 ± 39.3	0.315
HDL (mg/dL)	58.6 ± 12.5	57.1 ± 15.0	0.735
LDL (mg/dL)	122.8 ± 35.8	126.6 ± 27.9	0.702
Drug Therapies			
Antiplatelets, n (%)	3 (11%)	3 (19%)	0.655
Angiotensin II receptor blockers, n (%)	3 (11%)	2 (13%)	1.000
Angiotensin-converting enzyme inhibitors, n (%)	5 (19%)	8 (50%)	0.043
Calcium channel blockers, n (%)	1 (4%)	-	-
Beta-blockers, n (%)	9 (33%)	4 (25%)	0.735
Statins, n (%)	3 (11%)	3 (19%)	0.655
Echocardiographic data			
LVEF (%)	61.8 ± 6.4	67.1 ± 4.0	0.002
Left Ventricular Diastolic Volume (mL)	148.9 ± 42.6	141.1 ± 41.5	0.556
Left Ventricular Systolic Volume (mL)	56.3 ± 16.9	46.1 ± 13.0	0.033
Left Atrial Area (cm2)	29.8 ± 7.4	24.9 ± 4.9	0.015
PAPs	35.5 ± 8.9	32.9 ± 5.9	0.281
EROA (cm2)	0.4 ± 0.1	0.5 ± 0.2	0.112

Values are mean ± SD or n (%). BW: Barlow's disease patients; FED: fibro-elastic deficiency patients; LVEF: left ventricular ejection fraction; PAPs: pulmonary artery systolic pressure; EROA: effective regurgitant orifice area.

Supplementary Table S3. TaqMan probe assays' ID.

Assay Name	Assay ID
hsa-miR-140-3p	477908_mir
hsa-miR-150-5p	477918_mir
hsa-miR-210-3p	477970_mir
hsa-miR-223-3p	477983_mir
hsa-miR-27a-3p	478384_mir
hsa-miR-30c-5p	478008_mir
hsa-miR-323a-3p	477853_mir
hsa-miR-340-5p	478042_mir
hsa-miR-361-5p	478056_mir
hsa-miR-451a	478107_mir
hsa-miR-487a-3p	477826_mir
hsa-miR-186-5p	477940_mir

Supplementary Figures

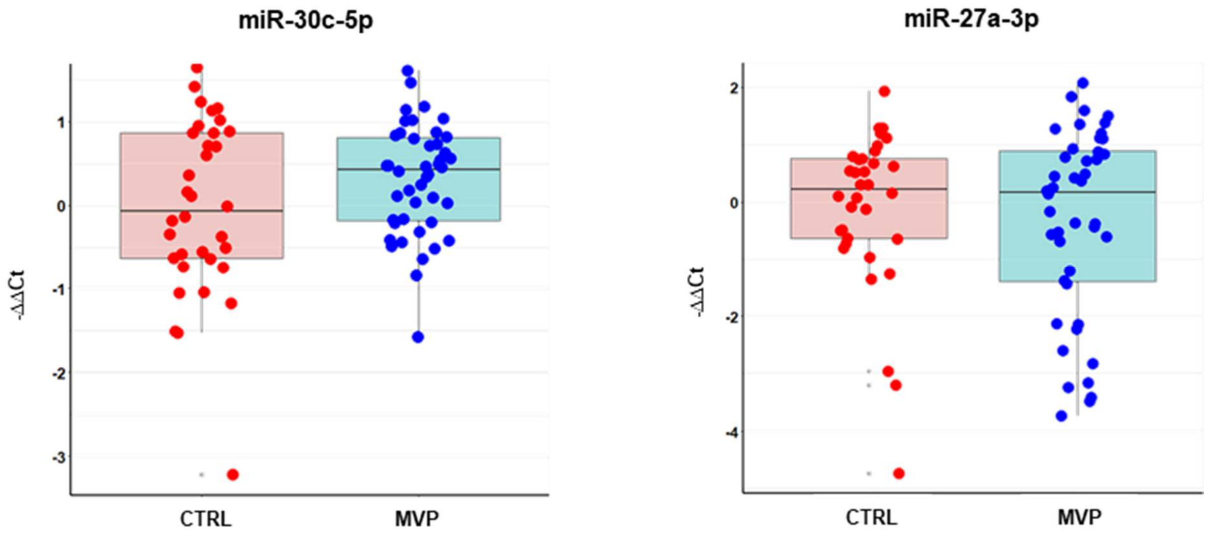

Supplementary Figure S1. Quantitative reverse transcription polymerase chain reaction validation. MiRNAs not differentially expressed between mitral valve prolapse patients (MVP, n = 43) and healthy subjects (CTRL, n = 34).

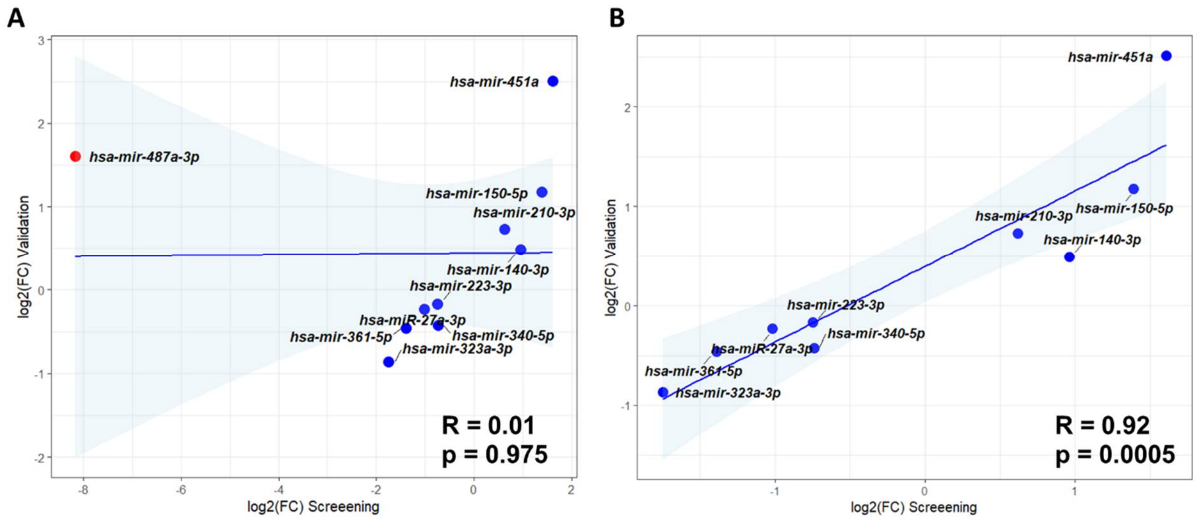

Supplementary Figure S2. Person's correlation of the log_2FCs for the validated miRNAs between the screening and validation phases. X-axis and y-axis represent the log_2FC of miRNA assessed on the screening and validation sets, respectively. Apart from miR-487a-3p (red dot in panel A), all validated miRNAs show the same expression ratio in the screening and validation datasets (panel B).
